# Mood Disturbances, Anxiety, and Impact on Quality of Life in Patients Admitted to Epilepsy Monitoring Units

**DOI:** 10.3389/fneur.2021.761239

**Published:** 2021-10-28

**Authors:** Rodrigo Rocamora, Beatriz Chavarría, Eva Pérez, Carmen Pérez-Enríquez, Ainara Barguilla, Luisa Panadés-de Oliveira, Alessandro Principe, Riccardo Zucca

**Affiliations:** ^1^Epilepsy Monitoring Unit, Department of Neurology, Hospital del Mar, Barcelona, Spain; ^2^Hospital del Mar Medical Research Institute, Barcelona, Spain; ^3^Faculty of Health and Life Sciences, Universitat Pompeu Fabra, Barcelona, Spain

**Keywords:** epilepsy, anxiety, depression, quality of life, epilepsy monitoring unit

## Abstract

**Introduction:** The overall combined prevalence of anxiety and depression in patients with epilepsy has been estimated at 20.2 and 22.9%, respectively, and is considered more severe in drug-refractory epilepsy. Patients admitted to epilepsy monitoring units constitute a particular group. Also, patients with psychogenic non-epileptic seizures can reach more than 20% of all admissions. This study aims to characterize these symptoms in a large cohort of patients admitted for evaluation in a tertiary epilepsy center.

**Materials and Methods:** The study was conducted among 493 consecutive patients (age: 38.78 ± 12.7, 57% females) admitted for long-term video EEG from January 2013 to February 2021. Demographic, clinical, and mood disorder patients' data were collected. Anxiety and depression symptoms were assessed through the Hospital Anxiety Depression Scale (HADS-A and HADS-D), the State Trait Anxiety Inventory (STAI), and Beck Depression Inventory (BDI-II). Quality of life was determined using the QOLIE-10. Patients were divided into three groups: patients with epilepsy (*n* = 395), psychogenic non-epileptic seizures (PNES) (*n* = 56), and combined (*n* = 33). A univariate and multivariate regression analysis was performed for variables associated with quality of life.

**Results:** Of 493 patients, 45.0% had structural etiology, and considering epilepsy classification, 43.6% were of temporal lobe origin. In addition, 32.45% of patients had a previous psychiatric history, 49.9% of patients had depressive symptoms in BDI, and 30.9% according to HADS-D; 56.42 and 52.63% of patients presented pathological anxiety scores in STAI-T and STAI-S, respectively; and 44.78% according to HADS-A. PNES and combined groups revealed a higher incidence of pathologic BDI scores (64.29 and 78.79%, *p* < 0.001) as well as pathologic HADS-A scores (*p* = 0.001). Anxiety and depression pathologic results are more prevalent in females, HADS-A (females = 50.7%, males = 36.8%; *p* = 0.0027) and BDI > 13 (females = 56.6%, males = 41.0%; *p* = 0.0006). QOLIE-10 showed that 71% of the patients had their quality of life affected with significantly higher scores in the combined group than in the epilepsy and PNES groups (*p* = 0.0015).

**Conclusions:** Subjective anxiety, depression, and reduced quality of life are highly prevalent in patients with refractory epilepsy. These symptoms are more evident when PNES are associated with epilepsy and more severe among female patients. Most of the cases were not previously diagnosed. These factors should be considered in everyday clinical practice, and specific approaches might be adapted depending on the patient's profile.

## Introduction

Anxiety and depression are frequent among patients with epilepsy (PWE) and constitute one of the most important comorbidities (1). Moreover, psychiatric disorders represent one of the principal modulating factors of the quality of life in PWE acting independently even of the seizure control itself (2). There is an agreement that principally depression but also anxiety are the main psychiatric comorbidities although the prevalence appears to be highly variable depending on the populations studied. However, the relevance of psychiatric comorbidities and the impact on quality of life in PWE has been consistent in different cultural contexts (3–5).

Contrary to the general concept of a higher prevalence of depression in PWE, recently it has been postulated that anxiety may be even more prevalent than depressive symptoms (6). Current analysis based on population studies in PWE described an overall prevalence of 23.1% for depression, and anxiety disorders ranged from 4.4 to 52.1% (7–9). On the other hand, when the studies carried out in patients with refractory epilepsy are analyzed, depression varies between 4.5 and 30%, and anxiety disorders are between 5 and 28% (10–13). A recent meta-analysis reports that the global pooled prevalence of anxiety disorders in PWE was 20.2%, and the overall pooled prevalence of depressive disorders was 22.9% (14).

Patients admitted for long-term video-EEG monitoring (LT-VEEGM) probably represent a different group. Drug-refractory epilepsy patients principally comprise them and less frequently differential diagnostic cases. Still, a significant percentage of patients admitted to an epilepsy monitoring unit (EMU) suffer from psychogenic non-epileptic seizures (PNES), and a non-negligible group presents an association of both pathologies. Diverse studies report that between 20 and 40% of patients admitted to an EMU suffer from PNES (15). In turn, ca. 9–11% of patients with PNES also present with epileptic seizures (16, 17).

Health personnel responsible for this group of patients are confronted not uniquely with epilepsies that are difficult to manage and also with a group of patients with psychiatric comorbidities probably differing from those reported in general population studies, which also determine their quality of life beyond epilepsy. In addition, the identification of psychiatric comorbidities is essential for defining multidimensional therapeutic strategies, preventing serious psychiatric complications and prognostic factors when making surgical decisions (6).

The objective of this study was to analyze the prevalence of anxiety and depression symptoms and to establish the impact on quality of life in a large sample of consecutive patients admitted for LT-VEEGM in our EMU.

## Materials and Methods

### Participants

The inclusion criteria for this study were admission to the EMU due to refractory epilepsy or differential diagnosis, aged 16 years or older, and having completed the evaluation tests. Exclusion criteria included intellectual disability (estimated IQ lower than 70), unwillingness to participate in the study, or insufficient proficiency in the Spanish language.

From January 2013 to February 2021, 836 patients were evaluated in the EMU of Hospital del Mar (Barcelona, Spain), a national reference center for refractory epilepsy and member of the European Reference Network EPI-Care. Finally, 493 consecutive (mean age: 38.78 ± 12.7 years, 57% females) patients were included in the study.

### Clinical and Sociodemographic Variables

Clinical data included medical records, seizure type, age at onset, seizure duration, current antiseizure medication (ASM) history, etiology, epilepsy localization, and history of psychiatric illnesses obtained from electronic clinical files and referenced by the patient. Sociodemographic data included age, gender, marital status, occupation, and educational level. Patients were subsequently classified into three groups: PWE (*n* = 395), PNES (*n* = 56), and combined (PWE + PNES, *n* = 33), and nine patients were not diagnosed and excluded from the analysis. These patient groups represent the totality of patients admitted to our EMU, which allows comprehensive research and may be helpful for comparison purposes.

### Epilepsy Variables

The group of PWE was analyzed to assess differences in the prevalence of psychiatric symptoms between focal vs. generalized epilepsies. Within focal epilepsies, prevalence in the temporal lobe vs. extratemporal epilepsy group was compared. The combined group was discarded from the analysis to avoid confounders.

### Psychiatric Assessment

The psychiatric evaluation was carried out using validated tests designed to measure levels of depression and anxiety. At the same time, the quality of life of the patients was assessed to evaluate the impact of the above symptoms on this important measure of well-being. Anxiety and depression symptoms were assessed through the Hospital Anxiety and Depression Scale (HADS), the State Trait Anxiety Inventory (STAI), and Beck Depression Inventory II (BDI-II) (18–21). Quality of life was determined using the Quality of Life in Epilepsy-10 (QOLIE-10), validated for the Spanish population (22, 23).

The PNES diagnosis was obtained after a consensual decision between epileptologists and psychiatrists. In our EMU, PNES are first detected by the epilepsy team as they occur during VEEGM. Immediately afterward, a referral psychiatrist with experience in epilepsy evaluates the behavioral aspects of the semiology and visits the patient during admission, establishing a specific treatment plan.

The HADS is a 14-item questionnaire designed to detect states of anxiety and depression symptoms in hospitalized patients. The HADS produces two scales, one for anxiety (HADS-A) and one for depression (HADS-D), and scores ≥8 on either scale indicate a pathologic case. The STAI is a 40-item, self-report scale assessing separate dimensions of “state” and “trait” anxiety. Items are rated on a 4-point Likert scale with higher scores indicating greater levels of anxiety. The BDI-II is a 21-item measure estimating the frequency and severity of depressive symptoms. Each item consists of four self-evaluative statements scored 0 to 3 with increasing scores indicating greater depression severity. The QOLIE-10 is a 10-item, self-report measure covering general and epilepsy-specific domains (medication effects, mental health, role functioning, and seizure worry) and scored on a 10 (normal) to 50 (very high) scale. BDI-II is the most widely used scale for detecting depression. Together with the HADS, it constitutes two of the three instruments approved by the National Institute for Health and Clinical Excellence to measure the severity of initial depression and response to treatment. Cutting scores for the different scales are reported in Table 1.

**Table 1:** Cutoff points of the inventory scales.

	**BDI-II**	**STAI-T**	**STAI-S**	**HADS-A**	**HADS-D**	**QOLIE-10**
Normal	0–13	0–20 (Males) 0–26 (Females)	0–20 (Males) 0–23 (Females)	0–7	0–7	10–20
Pathologic	>13	>20 (Males) >26 (Females)	>20 (Males) >23 (Females)	>7	>7	>20

### Ethics Committee

The protocol, informed consent, and any related relevant documents were examined and approved by the Clinical Research Ethics Committee (CEIC-Parc de Salut Mar). All patients signed an informed consent for the use of their data in this protocol. The study met the international and national good clinical practice as required by the principles of the Declaration of Helsinki of 2008 of the World Medical Association and the current legislation on protection of personal data (Organic Law 3/2018, of December 5, on the Protection of Personal Data and the Guarantee of Digital Rights).

### Data Analysis

The omnibus normality test (*scipy.stats.normaltest*) was carried out to examine the normality of the data. Chi-square and Fisher's exact statistics were used to compare proportions. Depending on the normality of data, *t*-test, Mann–Whitney, or Kruskal–Wallis tests for continuous variables were conducted to compare scores among the diagnostic groups. Multiple comparisons were corrected with Bonferroni adjustments. To determine the relationship between demographic, clinical, and mood factors and quality of life, a stepwise regression analysis on QOLIE-10 scores was conducted. The criteria for factor inclusion and exclusion were set at *p* = 0.01 and *p* = 0.05, respectively, and a list-wise deletion was used in the multivariate analysis. Statistical analyses were performed using SPSS21 (Armonk, NY, USA) and the scientific python library (*scipy*) with the level of significance set at *p* < 0.05 (two-sided) unless otherwise stated.

## Results

Of the population of 493 patients (57% female), 395 (80.12%) had epilepsy, 56 (11.36%) presented with PNES, and 33 (6.69%) had concurrent epilepsy and PNES (Tables 2, 3). Nine patients (1.83%) were not diagnosed and were not included in the group's comparison analyses. Of the total group with epilepsy (428 patients), 7.7% has PNES. Likewise, of the total group with PNES (89 patients), 37% has epilepsy.

**Table 2:** Clinical and sociodemographic characteristics of the patients included in the analysis (*N* = 493).

	**Total**	**Epilepsy**	**PNES**	**Combined**	***p*-value**
N, %	493 (100)	395 (80.12)	56 (11.36)	33 (6.69)	
Mean age at evaluation, years (SD)	38.78 (12.79)	38.55 (13.01)	39.98 (11.40)	40.03 (11.97)	0.68
Gender, *n* (%)					0.001
Females	281 (57.0)	207 (52.41)	43 (78.79)	25 (75.76)	
Males	212 (43.0)	188 (47.59)	13 (23.21)	8 (24.24)	
Marital status, *n* (%)					0.001
Single	222 (45.03)	195 (49.37)	14 (25.00)	9 (27.27)	
Married	216 (43.81)	162 (41.01)	35 (62.50)	16 (48.48)	
Widowed	8 (1.62)	6 (1.52)		1 (3.03)	
Divorced	39 (7.91)	25 (6.33)	6 (10.71)	7 (21.21)	
Couple	3 (0.61)	3 (0.76)	–	–	
Education, *n* (%)					0.32
Illiterate	3 (0.61)	3 (0.76)	–	–	
Primary	79 (16.02)	58 (14.68)	10 (17.86)	11 (33.33)	
Secondary	149 (30.22)	115 (29.11)	21 (37.50)	9 (27.27)	
Third cycle	145 (29.41)	119 (30.13)	15 (26.79)	7 (21.21)	
University	104 (21.10)	88 (22.28)	9 (16.07)	6 (18.18)	
Special education	5 (1.01)	5 (1.27)	–	–	
Occupation, *n* (%)					0.002
Employed	202 (40.97)	172 (43.54)	18 (32.14)	8 (24.24)	
Unemployed	155 (31.14)	116 (29.37)	21 (37.50)	16 (48.48)	
Retired	13 (2.64)	13 (3.29)	–	–	
Pensioner	77 (15.62)	52 (13.16)	15 (26.79)	8 (24.24)	
Student	41 (8.32)	38 (9.62)	1 (1.79)	1 (3.03)	
History of psychiatric disorders, *n* (%)					<0.001
Alcoholism	4 (0.81)	4 (1.01)	–	–	
Mood disorder	91 (18.46)	58 (14.68)	18 (32.14)	16 (48.48)	
Multiple	11 (2.23)	4 (1.01)	4 (7.14)	3 (9.09)	
No	316 (64.10)	285 (72.15)	18 (32.14)	9 (27.27)	
Not defined	6 (1.22)	2 (0.51)	2 (3.57)	1 (3.03)	
TOC	1 (0.20)	1 (0.25)	–	–	
Personality disorder	45 (9.13)	29 (7.34)	11 (19.54)	3 (9.09)	
Peri-ictal psychosis	2 (0.41)	2 (0.51)	–	–	
Number of ASMs, median (range)	3 (2, 3)	3 (2, 3)	2 (1–3)	2.5 (2, 3)	<0.001

**Table 3:** Clinical characteristics of the patients.

	**Epilepsy** **(*n* = 395)**	**Combined** **(*n* = 33)**	***p*-value**
Epilepsy etiology, *n* (%)			0.010
Genetic	15 (3.80)	–	
Structural/metabolic	209 (52.91)	13 (39.39)	
Unknown	96 (24.30)	6 (18.18)	
Epilepsy location, *n* (%)			<0.001
Generalized	17 (4.30)	–	
Frontal	72 (18.23)	2 (6.06)	
Insular	5 (1.27)	–	
Multifocal	16 (4.05)	–	
Unclassifiable	13 (3.29)	3 (9.09)	
Occipital	16 (4.05)	1 (3.03)	
Parietal	32 (8.10)	1 (3.03)	
Temporal	201 (50.89)	14 (42.42)	
Epilepsy onset age, mean (SD)	17.15 (12.83)	22.63 (14.57)	0.001
Seizure history in years, mean (SD)	21.24 (13.74)	17.86 (12.41)	0.001

Diagnostic groups were balanced for age (KW, *p* = 0.68) and education (chi^2^, *p* = 0.32), but not so regarding gender (chi^2^, *p* < 0.001 with females more prevalent in the PNES and the combined groups), epilepsy onset (KW, *p* = 0.001), epilepsy duration (KW, *p* = 0.001), marital status (chi^2^, *p* = 0.001), and employment (chi^2^, *p* =0.002). Patients' mean age at evaluation was 38.78 years (SD 12.79, 95% CI [37.65–39.91]), the mean age of epilepsy onset was 18.20 years (SD 13.52, 95% CI [16.98–19.43]), and the average duration of epilepsy was 20.43 years (SD 13.95, 95% CI [19.16–21.69]). Epilepsy duration was calculated as the interval (in years) from age at seizure onset to age at evaluation. A structural etiology was observed in 222 (45.0%) cases, and considering epilepsy classification, 215 (43.6%) were of temporal origin. In addition, 279 (61.18%) patients were on treatment with one or more ASMs with a median number of three [2–3] drugs. LEV was the most frequent ASM, being prescribed to 217 (44.0%) patients. Psychiatry disorders were previously diagnosed in 160 patients (32.45%) with mood disorders being the most prevalent (91 cases, 18.46%).

### Prevalence of Anxiety and Depression in the Total Population

A series of D'Agostino *K*^2^ tests revealed a non-normal distribution for QOLIE-10 (*K*^2^ = 15.962, *p* < 0.001), BDI-II (*K*^2^ = 45.720, *p* < 0.001), HADS-D (K^2^ = 26.718, *p* < 0.001), HADS-A (K^2^ = 21.766, *p* < 0.001), STAI-S (K^2^ = 16.324, *p* < 0.001), STAI-T (K^2^ = 29.839, *p* < 0.001).

**Figure 1:**
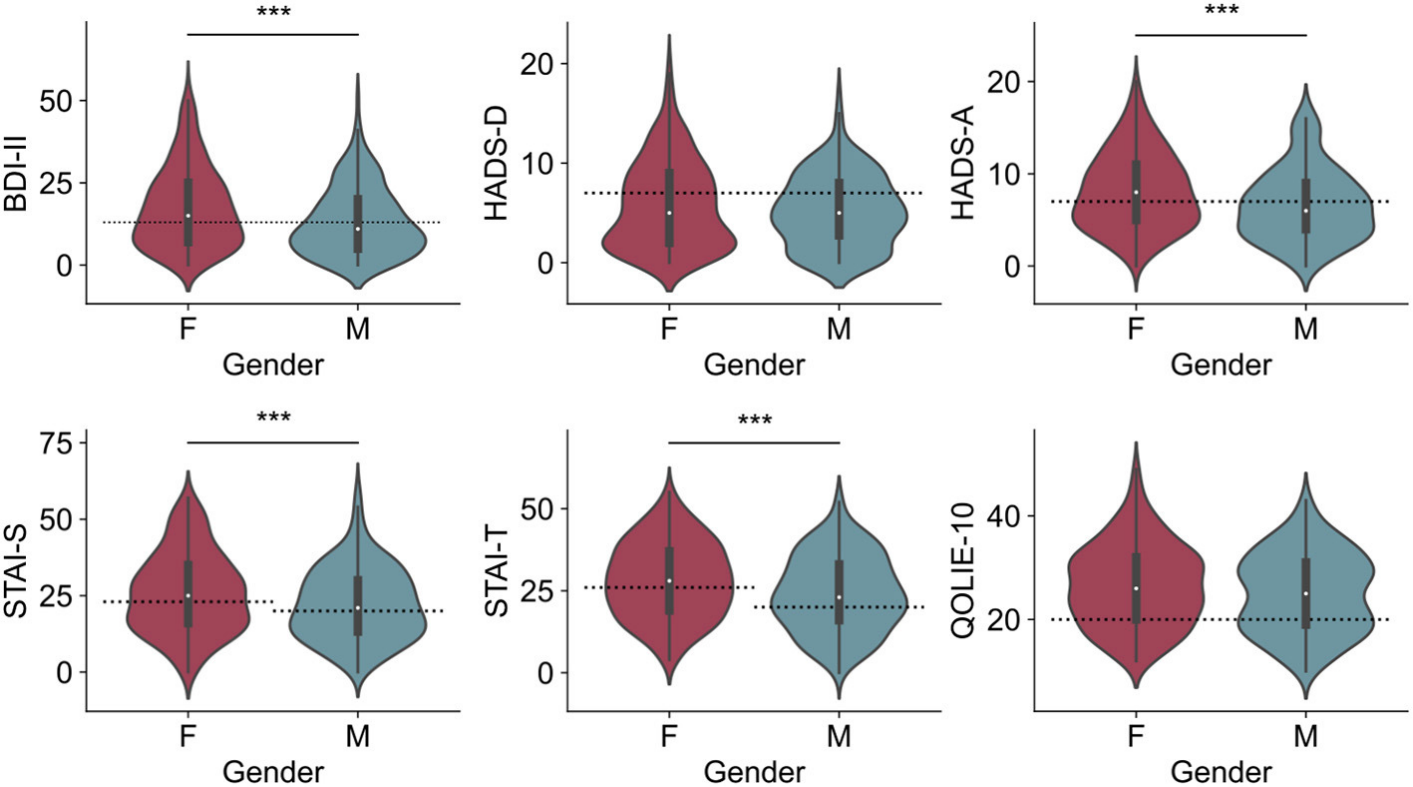
Median of scores on the inventory scales according to gender. BDI, HADS-A, STAI-S, and STAI-T scores were significantly higher in female patients. ***p < 0.01.

Depressive symptoms in the BDI-II (14 or above) were observed in 246/493 (49.90%) of the patients and 144/467 (30.84%) according to HADS-D (8 or above). The mean scores were 15.69 (SD 11.53) for BDI-II and 5.53 (SD 4.06) for the HADS-D scale (Tables 4, 5). Females had significantly higher BDI-II scores (females: 17.49, SD = 12.13; males: 13.31, SD = 10.24, *p* = 0.0002), whereas the difference was not significant for the HADS-D scores (females: 5.80, SD = 4.36; males: 5.19, SD = 3.59, *p* = 0.28) (Figure 1).

**Table 4:** Prevalence or frequency of depression, anxiety, and quality of life per diagnostic groups.

	**Total**		**Epilepsy**		**PNES**		**Combined**	
	**Normal**	**Pathologic**	**Normal**	**Pathologic**	**Normal**	**Pathologic**	**Normal**	**Pathologic**
BDI-II	247 (50.10)	246 (49.90)	215 (54.43)	180 (45.57)	20 (35.71)	36 (64.29)	7 (21.21)	26 (78.79)
STAI-T	214 (43.58)	277 (56.42)	181 (46.06)	212 (53.94)	22 (39.29)	34 (60.71)	8 (24.24)	25 (75.76)
STAI-S	216 (47.37)	240 (52.63)	184 (50.27)	186 (49.73)	21 (43.75)	27 (56.25)	9 (30.00)	21 (70.00)
HADS-A	259 (55.22)	210 (44.78)	222 (58.73)	156 (41.27)	23 (46.00)	27 (54.00)	5 (55.56)	4 (44.44)
HADS-D	323 (69.16)	144 (30.84)	270 (71.62)	107 (28.38)	34 (69.39)	15 (30.61)	11 (34.38)	21 (65.62)
QOLIE-10	139 (28.60)	347 (71.40)	119 (30.51)	271 (69.49)	13 (23.64)	42 (76.36)	5 (15.62)	27 (84.38)

**Table 5:** Average scores on anxiety, depression, and quality of life inventories per diagnostic groups.

	**Total**		**Epilepsy**		**PNES**		**Combined**	
	***N***	**Mean (SD)**	***N***	**Mean (SD)**	***N***	**Mean (SD)**	***N***	**Mean (SD)**
BDI-II	493	15.69 (11.53)	395	14.42 (11.01)	56	19.69 (11.77)	33	24.88 (12.18)
STAI-T	491	26.36 (11.66)	393	25.33 (11.48)	56	28.75 (12.33)	33	34.06 (9.83)
STAI-S	456	24.25 (12.65)	370	23.21 (12.38)	48	26.91 (13.20)	30	31.43 (12.06)
HADS-A	469	7.52 (4.09)	378	7.16 (4.03)	50	8.04 (3,95)	32	10.68 (3.71)
HADS-D	467	5.53 (4.06)	377	5.26 (3.81)	49	5.75 (4.71)	32	8.71 (4.30)
QOLIE-10	486	25.90 (7.83)	390	25.37 (7.74)	55	27.11 (7.71)	32	30.12 (7.59)

Pathologic anxiety scores in the STAI-S and the HADS-A were present in 240/456 (52.63%) and 210/469 (44.78%) patients, respectively. The mean STAI-S score was 24.25 (SD 12.65), whereas the mean HADS-A score was 7.52 (SD 4.09). Females had significantly higher anxiety scores in both the STAI-S (females: 25.86, SD = 13.11; males: 22.16, SD = 11.71, *p* = 0.003), and in the HADS-A inventory (females: 8.16, SD = 4.15; males: 6.66, SD = 3.87, *p* < 0.001) (Figure 1).

### Differences in the Prevalence of Anxiety and Depression Scores per Diagnostic Group

In our series, 360 patients had focal epilepsy (73.0%), and 17 were generalized (3.44%). Within the group of focal epilepsies, 215 (59.7%) were categorized as temporal lobe epilepsy (TLE), and 128 (35.5%) were grouped within the extratemporal group (Table 3). Across TLE and extratemporal epilepsies, no difference in the prevalence of pathologic scores was found for all the scales employed (all *p* > 0.05).

**Figure 2:**
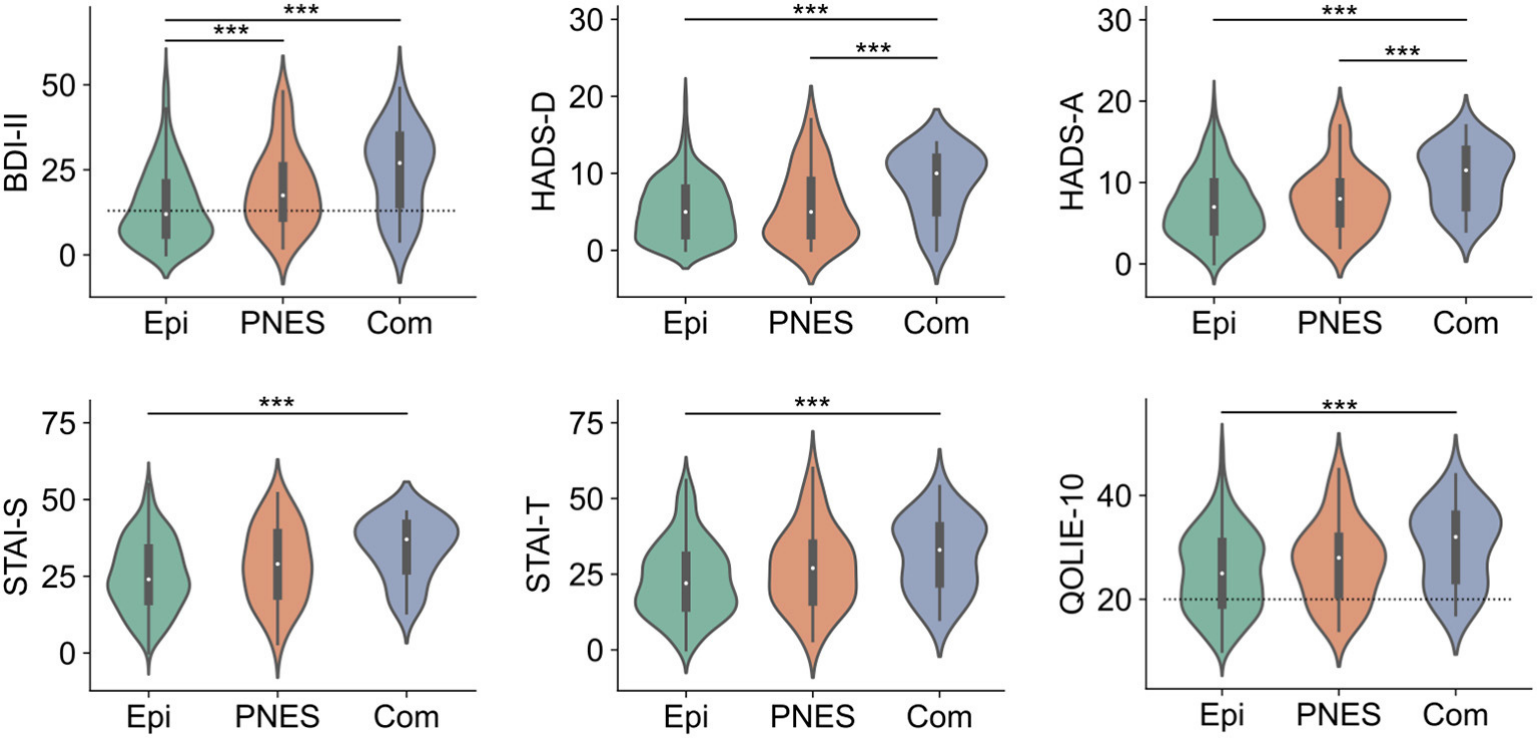
Inventory scores according to the diagnostic group. Epi, epilepsy group; PNES, psychogenic non-epileptic seizure group; Com, combined group. ***p < 0.01.

The PNES and combined groups revealed a higher incidence of pathologic BDI-II scores (64.29 and 78.79%, *p* < 0.001) as well as pathologic HADS-A scores (*p* = 0.001). The combined group showed a higher incidence of pathologic HADS-D scores (65.62%, *p* < 0.001). Pathologic anxiety and depression results were more prevalent in females, HADS-A (females = 50.7%; males = 36.8%; *p* = 0.0027) and BDI-II>13 (females = 56.6%; males = 41.0%; *p* = 0.0006) (Figure 2), but no gender differences could be observed for the HADS-D and STAI scales. A significant difference in the number of ASMs was observed for the PNES group, which, on average, was on less medication (*p* < 0.001).

### Quality of Life (QOLIE-10)

According to the QOLIE-10 scores (21 or above), 347/486 patients (71.40%) had their quality of life affected. The mean QOLIE-10 score was 25.90 (SD 7.83) with females showing a slightly higher incidence of pathologic QOLIE-10 scores (females = 74.73%, males = 66.99%, *p* = 0.06) (Figure 1). No significant difference in the prevalence of pathologic scores was found across diagnostic groups (*p* = 0.13) (Figure 2).

**Figure 3:**
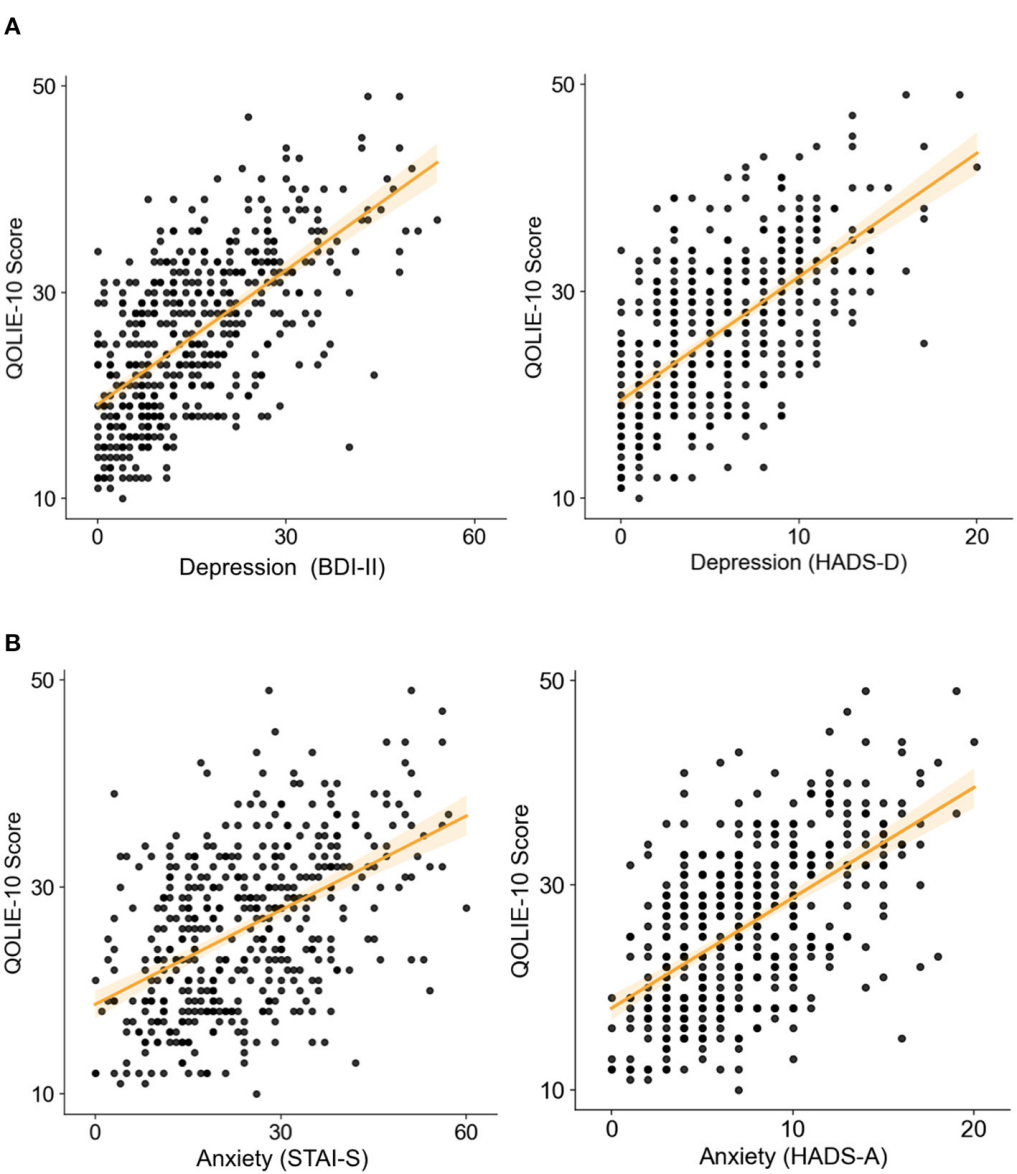
Inferior health-related quality of life is significantly associated with increased symptoms of depression and anxiety (higher score means lower perceived quality of life). Correlation between Quality of Life in Epilepsy Inventory-10 (QOLIE-10) overall score and scores of **(A left)** Beck Depression Inventory-II (BDI-II), **(A right)** Hospital-Anxiety and Depression-Scale (HADS) depression subscale, **(B left)** State-Trait-Anxiety-Inventory (STAI), and **(B right)** HADS anxiety subscale (all with *p* < 0.001).

Significant bivariate relations were observed between QOLIE-10 scores and the measures of depression, including the BDI-II and the HADS-D scale (*R*^2^ = 0.399 and *R*^2^ = 0.374, respectively, both *p* < 0.001). Increased endorsement of mood symptoms is associated with lower quality of life (Figure 3). A significant bivariate association was also observed between QOLIE-10 scores and measures of anxiety, including the HADS-A and the STAI-S inventories (*R*^2^ = 0.302 and *R*^2^ = 0.228, respectively, both *p* < 0.001). Increasing anxiety is similarly associated with a reduction in quality of life (Figure 3).

Depression and anxiety affect QoL independently. The partial correlations between QOLIE-10 and the two depression inventories remain significant after controlling for the two anxiety scales (BDI-II controlled for HADS-A, *R*^2^ = 0.163; BDI-II controlled for STAI-S, *R*^2^ = 0.223; HADS-D controlled for HADS-A, *R*^2^ = 0.396; HADS-D controlled for STAI-S, *R*^2^ = 0.199. All *p* < 0.001). Similarly, the partial correlations between the anxiety scores and the QOLIE-10 remain significant after controlling for the depression factor (HADS-A controlled for BDI-II, *R*^2^ = 0.054; HADS-A controlled for HADS-D, *R*^2^ = 0.077; STAI-S controlled for BDI-II, *R*^2^ = 0.043; STAI-S controlled for HADS-D, *R*^2^ = 0.036. All *p*s < 0.001).

We used multivariate stepwise regression to quantify the relative explanatory power of the different demographic, clinical, and mood factors on QOLIE-10 scores. The QoL score was significantly predicted (*R*^2^ = 0.477, *p* < 0.001) by a regression model, including the age of the patient, the age of epilepsy begins, the number of ASMs, depression (BDI-II, HADS-D scores), and anxiety scores (STAI-T) as latent factors.

## Discussion

Patients admitted to epilepsy monitoring units constitute a group of patients with unique characteristics. The presence of drug resistance and clinical features allow grouping and differentiating them even from outpatients, especially considering the diagnostic context. The admissions usually last a week in adult patients although they tend to be shorter for children. Admission times for invasive epilepsy procedures are even longer, lasting 2 or 3 weeks. During this time frame, medical efforts aim to answer epileptological questions, and subtle psychiatric disorders are usually overlooked (24). Throughout this period, patients are seen by nurses, medical technologists, neurologists, neurophysiologists, and health care teams indirectly related to epilepsy for whom the awareness of specific comorbidities is perhaps even more unknown.

Taking into consideration psychiatric comorbidities during the diagnostic process and subsequent discharge is relevant from several aspects. When the health care team is aware of psychiatric symptomatology, it can help establish a more empathetic physician–patient relationship and improve the patient's compliance to receive instructions, understand specific diagnostic procedures, and describe symptoms that psychiatric modulators can influence. In our series, 32.45% of the patients had a history of psychiatric mood disorders. The prevalence at the time of admission was higher, being between 30.84 and 49.9% for depressive symptoms, according to the scale used, and between 44.78 and 52.63% for anxiety symptoms. This finding is consistent with previous reports describing that psychiatric disorders are underdiagnosed in epilepsy (9). The determination of psychiatric comorbidity should influence a change in the choice of ASM with suitable characteristics for this comorbidity and the eventual indication of a specific psychiatric treatment under the specialist's control. Severe psychiatric symptoms, especially suicide risk, must be detected to establish adequate preventive measures (25, 26). On the other hand, the severity of the preexisting psychiatric pathology can be seriously affected after surgical procedures when this comorbidity is overlooked (27).

In our sample, we did not find a significant correlation between the number of ASMs and the prevalence of psychiatric symptoms or quality of life. However, other groups, using specific tools such as the Epitrack, a test specifically designed to evaluate cognitive side effects of medication, have found a negative correlation between them and the number of ASMs in TLE patients (28). A significant difference was only observed in the number of ASMs for the PNES group, which, on average, was on less medication. In the same line, other groups have found similar differences in this regard (24).

Several studies analyze the prevalence of psychiatric symptoms by epilepsy subtype. Some studies show a higher prevalence of mood disorders in TLE, arguing the involvement of mesial temporal structures part of the limbic system (29–31). However, many other studies find no differences during their lifetime (3, 32, 33). Methodological factors could explain these discrepancies. Various diagnostic instruments are used for psychiatric evaluation, ranging from questionnaires to more objective and reliable clinical diagnostic assessments. On the other hand, the diagnostic criteria for focal epilepsies can be inhomogeneous, depending on the setting in which the patient is evaluated. Finally, another confounding factor may be the use of diverse ASMs that can, in turn, modulate psychiatric factors in patients. The psychiatric findings of the studies are, therefore, difficult to compare (32). In our series, no differences were found between the prevalence of symptoms of depression or anxiety in TLE vs. extratemporal focal epilepsies despite the large number of patients evaluated. Nor were significant changes seen in the comparison between generalized and focal epilepsies. Only the duration of epilepsy in the PWE group was a risk factor for the appearance of symptoms. Therefore, our results support the hypothesis of a multifactorial cause in patients with refractory epilepsy (24).

Another relevant finding of this study is the gender differences found in the prevalence of psychiatric symptoms. The analysis of our series reveals that the BDI-II scores showed significant differences; that is, females had a higher incidence of pathological BDI-II scores than males (F: 56.58%, M: 41.04%). However, the comparison of HADS-D scores was not significant. Similarly, anxiety domains showed differences in pathologic HADS-A scores. Female patients revealed a higher incidence of pathologic HADS-A scores (50.75%) than male patients (36.82%) and scored higher also in STAI-S. Considering differences in quality of life by gender, a significant gap was also observed. Females (74.91%) had higher pathologic QOLIE-10 scores than males (66.5%). Recent studies also report similar results, suggesting that gender-specific approaches can be taken (34).

Besides epilepsy, a substantial number of the patients admitted to EMUs present with PNES, and a smaller group displays an association of both pathologies (35, 36). It is shown that PNES patients manifest functional, anatomical, and autonomic brain changes compared with healthy subjects and epilepsy patients without PNES (37–40). On the other hand, it is suggested that a wide range of psychopathologies may be the basis of PNES and that their treatment could improve clinical outcomes, avoiding the perpetuation of ongoing psychogenic seizures (35). In our series, 11.36% of patients presented isolated PNES, and another 6.69% had concurrent epilepsy and PNES, the total prevalence of PNES was 18.05%, which is consistent with previous reports (24, 33, 41). Of the total group with epilepsy (428 patients), 7.7% had PNES. Likewise, of the total group with PNES (89 patients), 37% had epilepsy. A recent metanalysis shows that the pooled frequency of epilepsy among those with PNES was 22% compared with 12% of PNES among those with epilepsy (42). In other words, in our case of EMU patients, the prevalence of epilepsy in PNES is approximately double among PWE, and that of PNES in PWE is around half. This could be explained by more selective screening of patients by excluding PNES before admission to UMEs compared with the general epilepsy population.

Furthermore, most studies exclude the mixed pathology group from their analyses. However, in our experience, it constitutes a clinical entity differentiated from patients with epilepsy or PNES alone. Our results suggest it is relevant to analyze this group separately.

When considering the prevalence of psychiatric symptoms in patients admitted to UMEs, a recent study of 101 patients detected that PNES patients scored significantly higher on the depression and anxiety scales than PWE. In addition, the overall QOLIE-31 score was worse for PWE than for PNES (3, 24). A different study including 200 participants shows that PNES patients have higher self-reported anxiety and depression levels but similar QoL to PWE (24).

In our comparative group analysis of depression rating scales, we observed that the PNES and combined groups have a higher incidence of pathologic BDI-II scores. BDI-II scores were significantly higher in the PNES (64.29%) and combined groups (78.79%) than the epilepsy group (45.57%). Similarly, HADS-D scores were significantly higher in the combined group (65.62%) than the epilepsy group (28.38%) and the PNES group (30.61%).

In relation to anxiety scores between the groups, a significant association of the pathological HADS-A scores is demonstrated, showing that the PNES and the combined group also have a higher incidence of pathological scores than the epilepsy group. HADS-A scores were also significantly higher in the combined group (71.88%) than in the PNES group (54%) and the epilepsy group (41.27%). On the other hand, The STAI-T score was significantly higher in the combined group (75.76%) than in the epilepsy group (53.94%) as were the STAI-S scores (70 and 50.27%, respectively).

Finally, these differences also corresponded with worsening in the quality of life of the patients. QOLIE-10 scores positively correlate with BDI-II scores and STAI-T. Partial correlations revealed significant independent relations between anxiety and depression and QoL, suggesting that the quality of life is affected similarly by both symptoms. Using multiple regression procedures, we also found that psychiatric comorbidities are relevant latent predictors of QoL associated with the patient's age, the age at which epilepsy was first diagnosed, and the number of ASMs. In the comparative analysis of groups, the QOLIE demonstrated pathological values in the group with epilepsy, PNES, and combined of 69.49, 76.36, and 84.38%, respectively. QOLIE-10 scores were also significantly higher in the combined group than in the epilepsy group.

These data confirm that patients with PNES have higher rates of depression than patients with isolated epilepsy, which has been previously reported (24). Moreover, we also found that patients suffering from both pathologies (epilepsy + PNES) present even higher ranges of depression and anxiety than patients with isolated psychogenic seizures or epilepsy. To interpret this difference, we propose a perspective within a broader framework, that is, a dual pathological model of the functional substrates of PNES and focal epilepsy. There is growing evidence from biomarker studies in PNES, suggesting that structural and functional changes observed in the brain may act as predisposing or precipitating factors for PNES. These changes could be secondary to early emotional trauma (37, 43). On the other hand, modern concepts of focal epilepsy interpret epileptogenicity based on the interaction of abnormal brain networks (44). How both etiological substrates interact is unknown, but they could theoretically explain the differences observed in the prevalence of psychiatric phenomena.

Our study has several limitations. First, it is a monocentric study with the constraints that this entails. Second, the scales used are for general psychiatric use and have not been designed explicitly for epilepsy. For this, using specifically developed scales, such as EpiTrack or the Neurological Disorders Depression Inventory for Epilepsy, could have provided more specific data. Third, we have not controlled the evolution of the patients, which could have provided important information regarding prognostic factors. Finally, our sample is based on the prospective collection of psychiatric symptoms using scales properly validated in Spanish but does not include clinical psychiatric diagnosis obtained through a specialized medical evaluation. Neuropsychiatric tests can identify people with anxiety and depression, but the results may be inconsistent with the clinical psychiatric evaluation. False negative screening tests can incorrectly assure that patients do not have a depressive or anxiety disorder, especially in patients with PNES (45). In our sample, only patients with PNES received, per protocol, a formal psychiatric evaluation at the time of VEEGM.

In conclusion, our study comprehends a large record of patients admitted to EMUs. Anxiety and depression symptoms are present in at least half of them with a direct negative effect on the quality of life of patients. Even more, anxiety symptoms seem to be more prevalent than depression. It provides valuable information comparing diagnostic groups, revealing that patients who have epilepsy associated with PNES present the highest rates of depression and anxiety. In addition, our analysis confirms that female patients show severer symptomatic and a worse QoL. Finally, it is evidenced that both depression and anxiety symptoms can independently affect the QoL of patients.

## Data Availability Statement

The raw data supporting the conclusions of this article will be made available by the authors, without undue reservation.

## Ethics Statement

The studies involving human participants were reviewed and approved by the Clinical Research Ethics Committee (CEIC-Parc de Salut Mar). The patients/participants provided their written informed consent to participate in this study.

## Author Contributions

RR was responsible for the study concept, methodology design, and medical writing. EP and AB were responsible for data collecting. BC was responsible for data collecting and statistical analysis. CP-E was responsible for data collecting, manuscript reviewing, and neuropsychological analysis. LP was responsible for data collecting and manuscript reviewing. AP contributed with data collecting and manuscript reviewing. RZ contributed with the statistical analysis, methodology, medical writing, and reviewing the article. All authors contributed to the article and approved the submitted version.

## Funding

This work received support from the project Emergent Cluster of the Human Brain (CECH) and the European Regional Development Fund under the framework of the ERFD Operative Program for Catalonia.

## Conflict of Interest

The authors declare that the research was conducted in the absence of any commercial or financial relationships that could be construed as a potential conflict of interest.

## Publisher's Note

All claims expressed in this article are solely those of the authors and do not necessarily represent those of their affiliated organizations, or those of the publisher, the editors and the reviewers. Any product that may be evaluated in this article, or claim that may be made by its manufacturer, is not guaranteed or endorsed by the publisher.
